# Serum Free Immunoglobulins Light Chains: A Common Feature of Common Variable Immunodeficiency?

**DOI:** 10.3389/fimmu.2020.02004

**Published:** 2020-08-11

**Authors:** Kissy Guevara-Hoyer, Juliana Ochoa-Grullón, Miguel Fernández-Arquero, Mariacruz Cárdenas, Rebeca Pérez de Diego, Silvia Sánchez-Ramón

**Affiliations:** ^1^Department of Immunology, IML and IdSSC, Hospital Clínico San Carlos, Madrid, Spain; ^2^Department of Immunology, Ophthalmology and ENT, School of Medicine, Complutense University of Madrid, Madrid, Spain; ^3^Immunodeficiency Interdepartmental Group (GIID), Madrid, Spain; ^4^Clinical Analysis Department, Hospital Clínico San Carlos, Madrid, Spain; ^5^Laboratory of Immunogenetics of Human Diseases, IdiPAZ Institute for Health Research, Madrid, Spain

**Keywords:** common variable immunodeficiency, serum-free immunoglobulins light chains, diagnostic tool, prognostic biomarkers, primary immunodeficiencies

## Abstract

Serum free light chain (sFLC) is a recently proposed biomarker for CVID diagnosis. Most CVID patients present low or undetectable sFLC up to 10-fold lower compared to other primary antibody deficiencies. Given that κ and λ light chains are normally secreted in excess with respect to immunoglobulins, this finding points to an intrinsic defect of B cell differentiation in CVID. sFLC levels were prospectively evaluated in a cohort of 100 primary immunodeficiency (PID) patients and in 49 patients with secondary immunodeficiency to haematological malignancy (SID). CVID patients had significantly lower κ and/or λ values (mean: κ: 1.39 ± 1.7 mg/L and λ: 1.97 ± 2.24 mg/L) compared to “other PIDs” (κ: 13.97 ± 5.88 mg/L and λ: 12.92 ± 7.4 mg/L, respectively, *p* < 0.001 both), and SID (κ 20.9 ± 22.8 mg/L and λ 12.8 ± 8.7 mg/L, respectively, *p* < 0.001 both). The sum of kappa and lambda (sum κ + λ) in CVID patients (7.25 ± 7.90 mg/L) was significantly lower respect to other PIDs (26.44 ± 13.25 mg/L, *p* < 0.0001), and to SID patients (28.25 ± 26.24 mg/L, *p* = 0.0002). ROC analysis of the sum κ + λ disclosed an area under the curve (AUC) of 0.894 for CVID diagnosis (SD 0.031; 95% CI: 0.83–0.95, *p* < 0.0001), with optimal cut-off of 16.7 mg/L, giving the highest combination of sensitivity (92%), specificity (75%) and NPV (98%). The Relative Risk (RR) for patients presenting a sum κ + λ below 16.7 mg/L was 20.35-fold higher (95%, CI: 5.630–75.93) for CVID than below this threshold. A similar behavior of the sFLC in our CVID cohort with respect to previously published studies was observed. We propose a cut-off of sum κ + λ 16.7 with diagnostic application in CVID patients, and discuss potential specific defects converging in low or undetectable sFLC.

## Background: Immunoglobulin, The Master Key of Many Locks

Given the high clinical variability and immunological heterogeneity in clinical manifestations of common variable immunodeficiency (CVID), several researchers have proposed combinations of clinical and immunological biomarkers in order to refine the diagnosis and to provide more personalized follow-up and treatment strategies that may improve the prognosis of the individual patient (1–4). A recently proposed biomarker for CVID diagnosis is the quantification of serum free light chain (sFLC) (5–8).

**FIGURE 1:**
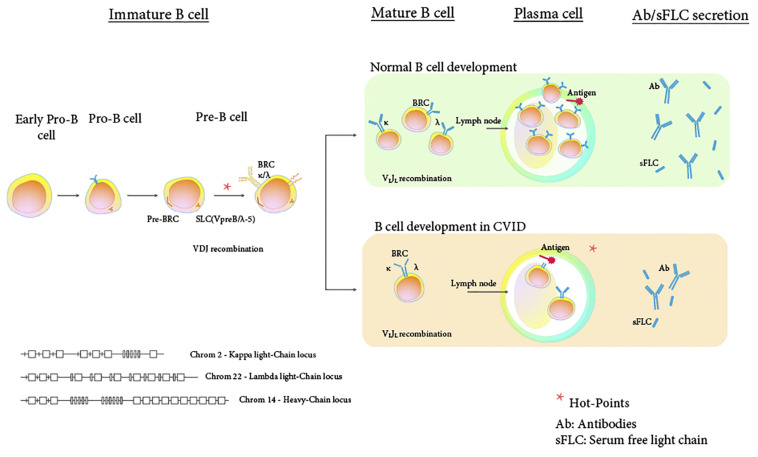
Potential hot-points in the development of sFLC synthesis, assembly and secretion. Scheme of the B cell maturation and differentiation, where an alteration in the rearrangement of the receptors of the pre-B cells, could condition the inadequate production of immunoglobulin light chains and, consequently, the defective expression of immunoglobulins. Modified from Winkler and Mårtensson (12).

The key-shaped structure of immunoglobulins (Ig) as originally described by Ehrlich (9), consists of four polypeptide chains, two pairwise identical copies of both heavy (H) and light (L) chains, the latter being named kappa (κ) or lambda (λ) chains (10). This “key” opens up a wide range of processes associated with innate and adaptive immunity. Among these processes stand out the direct neutralization of an almost unlimited number of antigens and toxins, autoantibodies, modulation of fas death receptor, binding to lectins, modulation of the complement cascade, regulation of monocytes/macrophages, activation of NK cells, regulatory T cell expansion, suppression of T and B cell activation, suppression of cytokines, neuroregulatory effects and increased sensitivity of steroids (10, 11). However, functions related to this “master key” are not completely known. L chains are incorporated into Ig molecules during B-cell development. Initially, large pre-B cells express a pre-BCR that is assembled from antibody μ H chains and surrogate L chain (VPREB1 and IGLL1). At the next stage, in small pre-B cells, bona fide L chains (κ and λ) undergo recombination and when this results in a productively recombined L chain, it is expressed together with μHC forming a BCR on the surface of pre B-cells (Figure 1) (12). Production in excess of L chains occurs throughout B-cell development till plasma cells, where they bind to H chains, excess L chains enter the bloodstream as FLCs. Secretion of L chains would reflect B cell activation (13, 14).

In healthy individuals, small amounts of both free L κ and λ chains can be found (κ = 3.3–19.4 mg/l, λ = 5.7–26.3 mg/l), with a normal κ/λ ratio ranging between 0.26 and 1.65 depending on the technical assay (5). These ranges were suggested using reference serum samples from 282 healthy donors between the ages of 21 and 90 years (15), based on the polyclonal Freelite assay.

sFLC quantification may indicate the presence of B cell clonality and is widely used in clinical practice for the diagnosis of B-cell lymphoproliferative disorders (B-CLPD), in particular the progression of monoclonal gammopathy of undetermined significance (MGUS) to multiple myeloma (MM), as well as a marker of neuroinflammation, for instance, in multiple sclerosis (16–23). Moreover, dysbalance in sFLC is used as a prognostic marker of various B-CLPD, such as chronic lymphocytic leukemia (CLL), B cell non-Hodgkin lymphomas (NHL), as well as for real-time monitoring of response to treatment and disease progression (5, 17, 24–27). Due to the inherent immunological alterations in a relevant proportion of PID patients, such as polyclonal B cell proliferation, it is particularly challenging to make an early diagnosis of B malignancy in these patients. Interestingly, alterations of sFLC in PID patients (κ/λ ratio), especially in CVID, correlate with clonal processes (6, 7, 13).

Here we sought to validate previous studies on the diagnostic and prognostic value of sFLC. Secondly, we suggest the sum κ + λ as a practical combined biomarker of CVID diagnosis and other potential applications for follow-up and prognosis. Finally, we present a hypothesis on the possible scenarios underlying very low sFLC in CVID and discuss potential experimental and clinical approximations.

## Materials and Methods

sFLC levels were prospectively evaluated in a cohort of 100 primary immunodeficiency (PID) patients and in 49 patients diagnosed with a hematological malignancy referred to study of secondary immunodeficiency (SID) at the Clinical Immunology Dept., Hospital Clínico San Carlos of Madrid, Spain. All PID patients fulfilled the ESID registry diagnostic criteria (28).

sFLC κ and λ chains were quantified by nephelometry (FREELITE, The Binding Site Group Ltd., Birmingham, United Kingdom), according to the manufacturer’s instructions, using a BNII nephelometer (Siemens Healthcare Diagnostics, Camberley, Surrey, United Kingdom).

SPSS statistics software (Chicago, IL, United States) was used for descriptive and statistical data analysis. Pearson’s correlation coefficient was used to assess the correlation between variables. *p* < 0.05 was considered statistically significant. Receiver operating characteristic curve (ROC curve) and contingency analysis were performed using GraphPad Prism version 8.3.0 for Windows, GraphPad Software, La Jolla, CA, United States^1^.

Approval for the study was obtained from the hospital institutional Ethics Committee for PID and SID projects (19-284-E and 19/219-E), respectively. Written informed consent was obtained from all patients for inclusion in the study protocol.

## Results

### sFLC Discriminates CVID From Other Primary Immunodeficiencies

**FIGURE 2:**
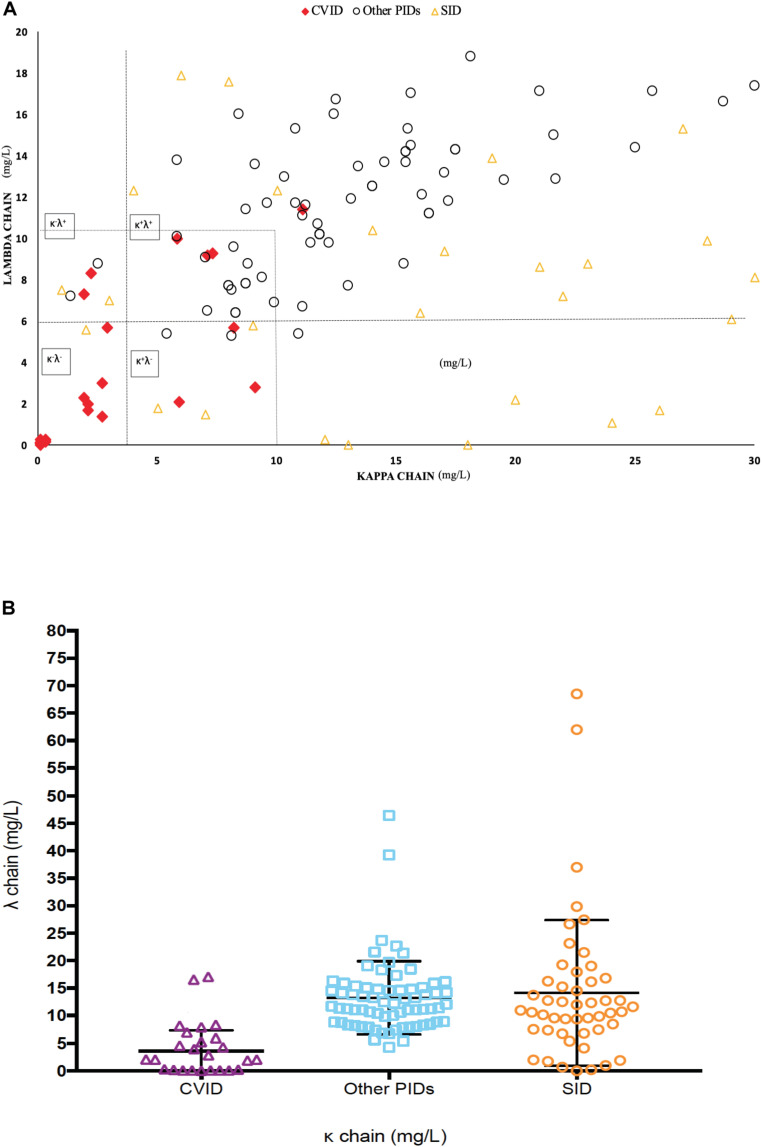
**(A)** Distribution of lambda and kappa chains among CVID, PID and SID patients (*N* = 100). **(B)** Distribution of sFLC concentration (sum κ + λ) in CVID *versus* other PIDs and SID.

We studied sFLC in 100 patients with different PIDs (selective IgA deficiency *n* = 38, unclassified antibody deficiency *n* = 27, CVID *n* = 26, Good syndrome *n* = 2, 22q11.2 deletion syndrome *n* = 2, complement system deficiency *n* = 2, X-linked hypogammaglobulinemia *n* = 1, hyper IgM syndrome *n* = 1, and Kabuki syndrome *n* = 1) as part as routine immunological work-up. CVID patients showed significantly lower κ and/or λ values in comparison to other PIDs (mean: κ: 1.39 ± 1.7 mg/L and λ: 1.97 ± 2.24 mg/L *versus* κ: 13.97 ± 5.88 mg/L and λ: 12.92 ± 7.4 mg/L, respectively, *p* < 0.001 both) (Figure 2). The sum of kappa and lambda (sum κ + λ) in CVID patients was 7.25 ± 7.90 mg/L versus 26.44 ± 13.25 mg/L respect to other PIDs (*p* < 0.0001).

When we analyzed the four previously described patterns of sFLCs in CVID (6–8, 29): 53.84% (14/26) disclosed the κ^–^λ^–^ pattern; 30.76% (8/26) the κ^+^λ^+^ pattern; 7.69% (2/26) the κ^–^λ^+^ pattern; and 7.69% (2/26) κ^+^λ^–^ pattern.

We then tested sFLC values in pure commercial gammaglobulin preparations, detecting mean levels of 4.15 mg/L of κ and 1.59 mg/L of λ, sum κ + λ 5.74 mg/L. However, it was insignificant when gammaglobulin was diluted 1:10, as found in normal plasma.

### sFLCs May Aid in the Diagnosis of Secondary Immunodeficiencies

Regarding the comparison of sFLC expression in SID and CVID patients, we evaluated 49 patients with hematological malignancy: CLL (*n* = 12), NHL (*n* = 22), MGUS (*n* = 12) and MM (*n* = 3). CVID patients showed significantly lower kappa and/or lambda values than SID (mean: κ 1.39 ± 1.7 mg/L versus κ 20.9 ± 22.8 mg/L, *p* < 0.001; and λ 1.97 ± 2.24 mg/L versus λ 12.8 ± 8.7 mg/L, respectively, *p* < 0.001). When comparing the κ/λ ratio in both cohorts, CVID κ/λ ratio was significantly lower than SID (0.94 versus 1.91, *p* < 0.005). The sum κ + λ in CVID was also significantly lower than SID patients (7.25 ± 7.90 mg/L versus 28.25 ± 26.24 mg/L, *p* = 0.0002).

In our SID cohort to B-CLPD, 7 patients with CLL and NHL that showed very low or undetectable sFLC at diagnosis of malignancy were highly suspicious of an underlying CVID based on history of infections since childhood (*n* = 2) or suspicious family history of PID (*n* = 2), which were not diagnosed at the time, which could only be confirmed if PID-predisposing gene defect were found (30).

### ROC Curves

The diagnostic performance of a CVID-pattern (i.e., diminished levels of free L κ and/or λ in respect to the reference range) was evaluated for the diagnosis of CVID with respect to other PID and SID, showing a sensitivity of 76.00%, specificity of 85.71%, positive predictive value (PPV) 52.78% and negative predictive value (NPV) of 94.4% for CVID diagnosis.

We then compared the diagnostic value of the sum of free L κ + λ levels in this setting. ROC analysis disclosed an area under the curve (AUC) of 0.894 (SD 0.031; 95% CI: 0.83–0.95, *p* < 0.0001), with optimal cut-off of 16.7 mg/L for the sum κ + λ giving the highest combination of sensitivity (92%) and specificity (75.6%), with NPV (97.8%). The Relative Risk (RR) found for CVID was of 20.35-fold (95% CI 5.630–75.93) for patients with sum κ + λ levels below 16.7 mg/L (Supplementary Figure S3). The frequency of patients below the recommended sum κ + λ cut-off of 16.7 mg/L was 92% of CVID patients; 10% of other PIDs; and 28% of SID (*p* < 0.0001, both).

### Comparison of sFLC Values With Clinical Associations in CVID

We observed a significant correlation between κ^–^λ^–^ pattern and CD27^+^IgD^–^IgM^–^ switched-memory B cells (*p* < 0.05). We did not find statistical significance between specific sFLC patterns and age (*p* = 0.20), sex (*p* = 0.30), clinical onset (*p* = 0.15), CD21^*low*^ B cells (*p* = 0.76) or Ig levels at diagnosis of CVID (*p* = 0.94). We then compared sFLC patterns (κ^–^λ^+^, κ^+^λ^–^, κ^–^λ^–^, κ^+^λ^+^) with clinical associations in CVID patients. We found that κ^–^λ^–^ pattern was highly prevalent in CVID patients with enteropathy (66.66%, 4/6 patients, *p* = 0.01), splenomegaly (50.00%, 7/14 patients, *p* = 0.2) and bronchiectasis (68.75%, 11/16 patients, *p* = 0.1), compatible with the association described by other groups (6, 7, 29).

Regarding clinical associations in CVID patients using the sum κ + λ below 16.7 versus above this cut-off, 28.57% (6/21 patients) presented enteropathy versus 0% (*p* = 0.1), 57.14% (12/21) splenomegaly versus 40% (*p* = 0.4) and 66.66% (14/21) bronchiectasis versus 40% (*p* = 0.2).

## Discussion

### sFLC as a Diagnostic Tool in Primary Immunodeficiencies

Compatible with previous studies, a similar distribution of normal sFLC levels among all PID was observed, except for CVIDs, with significantly lower κ and/or λ values than other PIDs (*p* < 0.001). To better define the most optimal cutoff level for sFLC in our population, we used the sum κ + λ of 16.7 through standard statistical ROC analyses for discriminating CVID from other PID and SID, AUC of 0.894 (*p* < 0.0001, for both). The sum κ + λ provided a high sensitivity, specificity and NPV for CVID diagnosis.

Several groups have pioneered a greater understanding on the role of sFLCs in the clinical scenario of PIDs (6–8, 29). While many primary antibody deficiency (PAD) share a common profile of hypogammaglobulinemia, it has not been fully elucidated why only CVID presents a characteristic profile of low or undetectable sFLC (6–8, 29). Unsworth et al. described very low sFLCs in 18 out of 20 PID cases, CVID being the commonest diagnosis (16/20), followed by X-linked agammaglobulinemia (XLA, 2/20). Hyper-IgM syndrome (HIGM, 1/20) and non-HIGM with raised polyclonal IgM (1/20) showed normal sFLC values. The common denominator for all 20 patients was antibody immunodeficiency and associated increased frequency of bacterial infections (8). Scarpa et al. have recently described significant variations in sFLCs values in a wide cohort of PID. CVID patients showed decreased or undetectable values of sFLCs compared to normal values in patients with unclassified antibody defects (*p* < 0.0001) (7). Their findings were similar to those described in other studies with different PIDs cohorts (6–8, 29, 31, 32). Regarding XLA, there are contradictory results in the different cohorts described, ranging from low to normal sFLC, which may depend on the genetic defect (33).

In the present study, we used nephelometry with the same test, reagents, and platforms described by Scarpa et al. and Unsworth et al. in PIDs cohorts (7, 8), which reduces the variability among results. The technique used has shown the highest sensitivity for sFLC, with detection up to 0.1 mg/L for both κ and λ as low reference levels (5). However, it could be interesting to design comparative studies between the different techniques currently available for measuring sFLC in the PID setting.

The marked decrease in sFLCs could reflect a profound damage, both quantitatively and functional of the BCR during lymphocyte differentiation in CVID. Moreover, a dysfunctional BCR might be at the origin of a lymphoproliferative or self-reactive status in this patients’ population (12). In general, the production of both L chains increases during infections or inflammatory states, with higher absolute concentrations but without change in κ/λ ratio (5, 6, 8). Infection and inflammation are very common and define the clinical phenotypes “cytopenia,” “polyclonal lymphocytic infiltration,” “unexplained enteropathy,” and “no disease-related complications (only infections)” of CVID patients (3). We could infer that CVID patients with chronic inflammatory phenotype would present with high levels in a κ^+^λ^+^ pattern. Scarpa et al. analyzed the association of sFLCs patterns (κ^–^λ^+^, κ^+^λ^–^, κ^–^λ^–^, κ^+^λ^+^) and CVID clinical phenotypes. Counterintuitively, infectious and inflammatory phenotypes were more frequently observed in CVID patients with low or absent levels of sFLC (κ^–^λ^–^) (6, 7, 29). Likewise, we found that enteropathy, splenomegaly and bronchiectasis were more prevalent in the κ^–^λ^–^ pattern in our cohort, although the non-significance in splenomegaly and bronchiectasis could be due to the small sample size (Supplementary Figure S1). Our findings did not demonstrate statistical significance between specific sFLC patterns and age, sex, clinical onset or Ig levels at diagnosis of CVID. In the study of Compagno et al., κ^–^λ^+^ pattern was the most represented in CVID (21 out of 46 patients, 46%) with higher risk of mortality derived from autoimmune cytopenias, lymphoproliferation and enteropathy (12/21 patients, 57%), followed by κ^–^λ^–^ pattern (15/46 patients, 33%) with a trend to present splenomegaly (6/15 patients, 40%) and malignancy (5/15 patients, 33%) (6). Scarpa et al. hypothesized that low sFLC levels may be an epiphenomenon of a higher degree of impairment in B cell differentiation, with reduced B cell class-switch affecting immunoglobulins’ production (7). Altogether, these findings support that diminished sFLC values observed in CVID are associated with this pathology and can be used as an accessory diagnostic tool to support CVID diagnosis. However, the clinical significance of these patterns is still under study and needs further validation.

### Comparison of sFLC Patterns With Other CVID Biomarkers

There is no clear correlation between sFLC with all serum Ig levels at diagnosis in the different PIDs groups studied (7, 8, 29). Scarpa et al. described a direct association of IgA and IgM with serum κ and λ chain concentrations in CVID but not in control groups, while no association between sFLC and serum IgG neither in CVID or control groups (7). Unsworth et al. did not find correlation between IgG and IgA values with sFLC concentrations in their PID cohort (8). Hanitsch et al. described significantly lower IgG levels in κ^–^λ^–^ CVID, although IgA and IgM levels were not different (29).

There are controversial results regarding sFLC with B cell phenotype in CVID patients. We observed a significant correlation between κ^–^λ^–^ pattern and class-switch CD27^+^IgD^–^IgM^–^ memory B cells (*p* < 0.05), without association between CD21^*low*^ B cells and sFLC patterns. In contrast, Compagno et al. described a significant decrease in numbers of switched memory, marginal zone, CD21^*low*^ B cells in the κ-λ- pattern, and a marked decrease of the subsets linked to B-cell activation and Ig production, while no correlation with transitional B cells (6). IN contrast, Scarpa et al. showed the highest frequency of CD21^*low*^ B cells in κ-λ- group (7). The clinical association derived from these results warrants further study.

Most of our patients were on Ig replacement therapy (IgRT), and serum testing at CVID diagnosis and pre-infusion. Commercial gammaglobulin preparations of pooled normal IgG did contain detectable κ and λ sFLCs, similar to previously published data (8), thus it seems unlikely that they may affect the results, since all patients had normal renal function and the half-life of sFLC in the circulation is 2–6 h (5). IgG infusions are typically repeated every 3–4 weeks so that pre-infusion concentrations measured are likely to only contain sFLC produced by the patient’s immune system. Likewise, the multi-time measurement of sFLC in order to determine intra-individual variability showed no difference in our patients (data not shown).

### sFLC and the Dilema Between Primary and Secondary Immunodeficiencies

There is a cancer-immune paradox in PID described by some authors (34). The type of malignancy seems to be highly dependent on the specific PID, the age of the patient, and chronic infectious stimuli or dysbiosis involving complex pathogenic mechanisms (35). Cancer is 1.4 to 5-fold higher in registry-based PID studies respect to general population (36, 37), from which 70% corresponds to lymphoid malignancy (38, 39). Individuals with CVID are at 5 to 10-fold higher risk of developing hematological malignancies (36, 37, 40), while unexpectedly lower incidence on most common cancers than general population has been described (41, 42). Cancer is a leading cause of mortality in PID, and thus early diagnosis and treatment of malignancy is a priority (22, 43). When comparing the κ/λ ratio in both cohorts, SID showed significantly higher κ/λ ratio (*p* < 0.005), as expected (7, 29, 43). In CVID patients, the κ/λ ratio is usually normal. In CVID, a B-CLPD can be the first and only clinical manifestation and thus the diagnosis of PID versus SID represents a difficult clinical dilemma. Low κ and/or λ values at hematological malignancy diagnosis might be pointing an underlying CVID. There are no data on the “potential PID patients” in the whole pool of patients with B-CLPD, which may justify to investigate an underlying PID as cancer predisposing factor (35, 40, 41, 44). Also, κ/λ ratio could be important in the follow-up of CVID patients, and hence an altered κ/λ ratio or a sudden increase in sFLC values may be an indicator for further investigation (blood smear, LDH, serum β2-microglobulin, PET-TAC, etc.) that allows appropriate and timely strategies. We consider that the κ/λ ratio behaves like a more reliable marker than the isolated determination of the sFLC when comparing both cohorts.

### The Key to CVID: Distinctive Light Chain Defect

The extremely low sFLC in CVID might be explained by different reasons: (i) the lowest the plasma cells numbers, the lowest secretion of sFLC; (ii) increased elimination of sFLC; (iii) altered rearrangement, assembly or secretion of LC during B cell ontogeny; (iv) an intrinsic defect of plasma cells secretion of light chains. To address precisely the first argument, we should quantify plasma cells in the bone marrow, which is not feasible. Indirect measures of total peripheral B cells, class-switched B cells or total serum IgG did not explain sFLC (Supplementary Figure S2). We discarded the second argument, since none of the patients had renal or other protein loss. The two last hypothesis imply that the low levels of sFLC in CVID patients may reflect an intrinsic alteration affecting normal production, assembly or secretion of L chains into Ig molecules, which points either to specific defects in plasma cell differentiation (45), or stretches the way back to a critical early event during B cell differentiation. Ig genes are first rearranged in early B cell development through the V(D)J recombination in the liver and then bone marrow and then further modified upon antigenic encounter through the somatic hypermutation (SHM) process in germinal centers of lymphoid nodes. We hypothesize that different underlying mechanisms might correlate with different sFLC patterns, which we discuss below according to clinical and immunological observations and experimental published data:

1. Early B cell defect: Particularly in cases where sFLC are undetectable, L chains should be more profoundly affected than H chains, for which a revision of genetic and molecular processes in the generation of Ig diversity is required. L chains of Ig are encoded in different multigene families from H chains and in different chromosomes: κ in chromosome 2p11.2, λ in chromosome 22q11.2 and H in chromosome 14q32.2, Several potential genetic variations might occur during this process (10, 31), affecting rearrangement of H and L chains in CVID (germline polymorphisms, allelic variants, insertion, deletion, etc.) (14, 46). At pre-B cells stage, first phase (antigen-independent) rearrangement of the H chain occurs, which reacts with light chain-like molecule called surrogate L (SL) chains by allelic exclusion (14, 46, 47). During the cell division cycles, the composition of the pre-B cell receptors (BCR) in the daughter cells engender successful production of a complete κ or λ light chain and further allows the expression of IgM on B cell surface (10).

The pre-BCR is a heterodimer composed of a H chain covalently associated with a surrogate light chains (SL) chain, a temporary common light chain composed by two non-covalently associated proteins, namely lambda-5 (λ5) and V-preB, which together have structural homology with conventional L chains (48). VDJ recombination of the H chain (pre-B cell) precedes pairing with SL chains, proliferation of large pre-B cells and subsequently L chain rearrangement (Figure 2). At this stage (at pre-BI to pre-BII or at pre-BII cell to immature B cells for pre-BCR), any transcriptional error in the SL chains, involving the region of the interchain bond during pre-B cells, would affect the correct linkage of SL to H chains and results in lack of LC. Conley reported the first patient with autosomal recessive mutation in the λ5 gene causing severe B cell deficiency and agammaglobulinaemia (33). The mutant λ5 resulted in impaired protein folding and secretion of Ig. It is conceivable that alteration of the players of this highly coordinated process at the pre-BCR stage may result in complete lack of secretion of L chains and undetectable sFLC. Isolated lack of single L chains occurs lately during this sequence. Complete absence of κ chain with normal Ig concentrations has been reported in a patient due to a heterozygous point mutation (1288 GG) that generated an amino acid substitution from Cys to Gly in the protein sequencing, causing an abnormal folding of the polypeptidic constant region of κ chain (31). In this patient, lack of κ chains determined a reduced antibody repertoire despite normal Ig concentrations, associated to recurrent bacterial infections. A correct class switch of H chains requires that the functional CH genes, located at one end of the rearranged H chain, are activated (10, 47). This synergy of mechanisms might explain in part why, in CVID patients, the decrease in sFLC is frequently associated with the inability to generate CD27^+^IgD^–^IgM^–^ switched memory B cells. Additionally, an aberrant recombination of the L chain gene repertoire might favor autoreactive phenomena (49), while altered DNA repair controlling this recombination process would entail an increased pool of aberrant protein involves in B-cell oncogenesis (50, 51).

2. Plasma cell defect: acquired or genetic functional B cell defects have been described after TLR, CD40 and BCR-mediated NFκB signaling pathways that account for altered memory B cell phenotype in CVID patients and low Ig (52, 53). Deep sequencing of IgH locus demonstrated restriction of characteristic patterns of IgHV and IgHJ usage depending on the B cell stimulus (54). Specific defects in the terminal plasma cell differentiation were shown in a subgroup of CVID patients at germinal center responses, with diverse mechanisms converging into the block of final step of plasma cell differentiation (45). In addition, gene lesions in MSH5 has been related to a few CVID patients, with defective S region junctions between LC and HC that would take place after antigen encounter (55). Altogether, these diverse mechanisms result in reduced and modulated class-switched B cells and Ig, which may relate to L chains’ restriction and low levels of sFLC secretion (Figure 1).

### Concluding Remarks and Further Perspectives

Our data validates previous studies emphasizing the relevance of sFLC quantification in the diagnosis and follow-up of CVID patients. sFLC behaves as a promising biomarker in the differential diagnosis of CVID with other PID and SID, and κ/λ ratio as a prognostic biomarker associated with specific clinical phenotypes. A cutoff level κ + λ < 16.7 mg/L supports CVID diagnosis. Moreover, κ/λ ratio alteration or a sudden increase in sFLC values may alert lymphoid malignancy and prompt appropriate and timely diagnostic work-out and therapy, a major concern in this patients’ population that impacts the survival. Reference values and cut-off points must be validated for each technique and then compare the different available immunoassays to come up with a reference range for each assay in different populations.

We hypothesize that decreased levels of sFLC in CVID patients may reflect an intrinsic early defect at a critical common step of B cell differentiation in the bone marrow affecting SL or L chain assembly or secretion that would affect memory B cell phenotype. Work is ongoing to check the hypothesis rooting this phenomenon by discarding gene defects during early B cell ontology, or intrinsic alterations in the terminal plasma cell differentiation, by *in vitro* differentiation of plasma cells from CVID patients after stimulation with subsequent determination of sFLC production and secretion. Altogether, we provide new evidence that this biologic phenomenon of low κ and λ provides a common feature of CVID, and leaves entirely open the question of whether would it be necessary to revisit the classification of CVID according to it.

## Data Availability Statement

The raw data supporting the conclusions of this article will be made available by the authors, without undue reservation.

## Ethics Statement

The studies involving human participants were reviewed and approved by the ethical committee of the San Carlos Clinical Hospital, Madrid, Spain. The patients/participants provided their written informed consent to participate in this study.

## Author Contributions

KG-H and SS-R designed the study, integrity and analysis of data, and writing of the manuscript. RP, JO-G, and MF-A contributed to the analysis of data. KG-H contributed to the design of the images and figures. MC contributed to the measurement of the serum-free light chain and the analysis of data. All authors reviewed and approved the final version of the manuscript.

## Conflict of Interest

The authors declare that the research was conducted in the absence of any commercial or financial relationships that could be construed as a potential conflict of interest.

## Footnotes

1 www.graphpad.com

## Supplementary Material

FIGURE S1 Bar chart comparing sFLCs patterns (κ^–^λ^+^, κ^+^λ^–^, κ^–^λ^–^, κ^+^λ^+^) with infectious and inflammatory phenotypes (Enteropathy, splenomegaly, bronchiectasis and cytopenia’s).

FIGURE S2 Scatter plots comparing Kappa light chain concentration against: **(A)** Serum IgG at diagnosis; **(B)** Class-switched Memory B cells (CD19 + IgD-IgM-CD27 +); **(C)** B cells CD19%.

FIGURE S3 Receiver operating characteristic (ROC) curves for CVID diagnosis with the sum κ + λ testing. The area under the curve (AUC) for CVID was 0.894 (*P* = 0.001). The use of sum κ + λ testing identified 24 of 26 cases of CVID, for a sensitivity of 92% [95% confidence interval (CI), 0.83–0.95 (*p* < 0.0001)].
