# Supplementary figures and images for: Serum Free Immunoglobulins Light Chains: A Common Feature of Common Variable Immunodeficiency?
Source: Front Immunol. 2020 Aug 11;11:2004. doi: 10.3389/fimmu.2020.02004 (PMC7431983; doi:10.3389/fimmu.2020.02004)

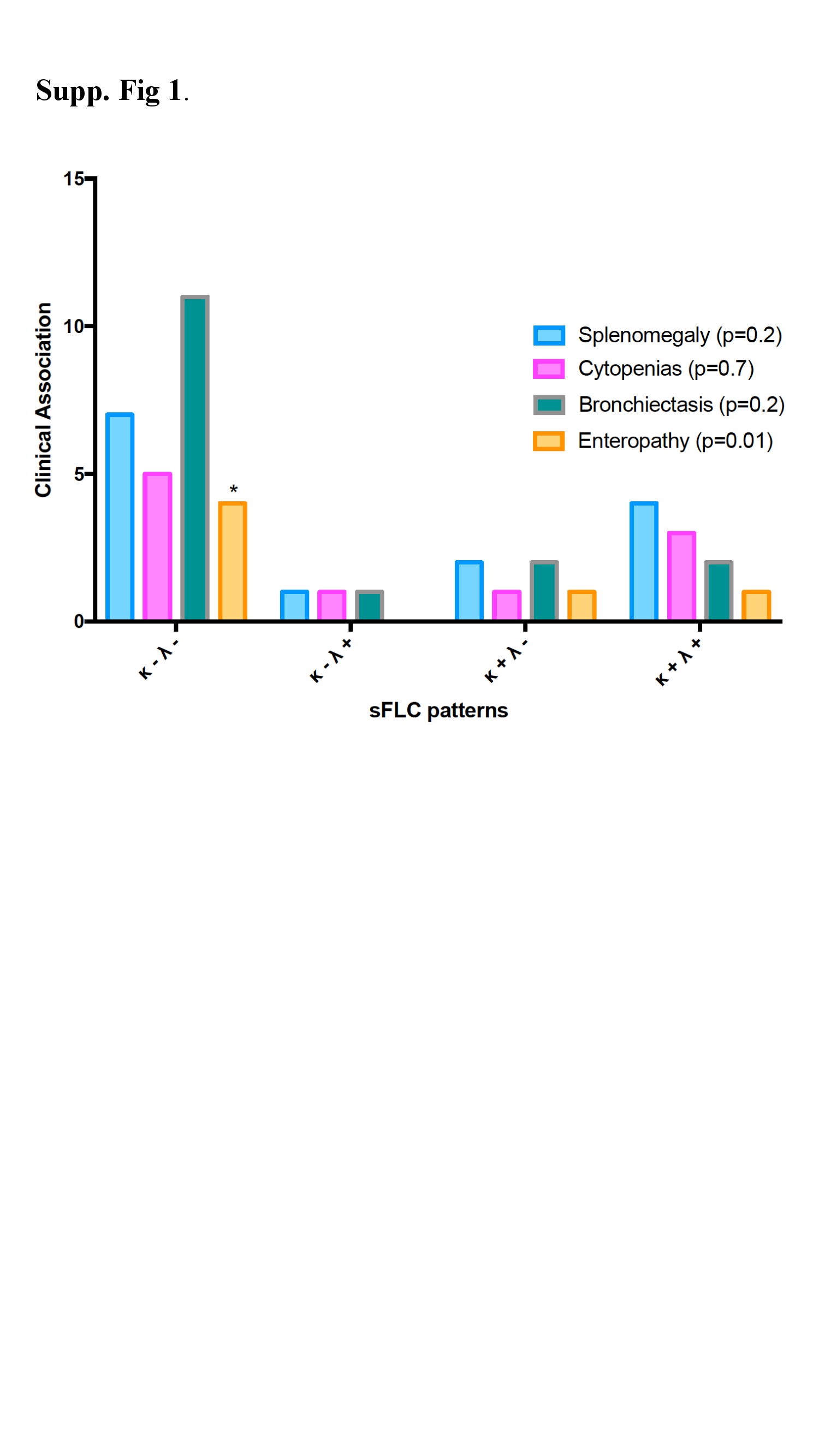

Supplement: FIGURE S1 — Bar chart comparing sFLCs patterns (κ–λ+, κ+λ–, κ–λ–, κ+λ+) with infectious and inflammatory phenotypes (Enteropathy, splenomegaly, bronchiectasis and cytopenia’s). [file Image_1.TIFF]

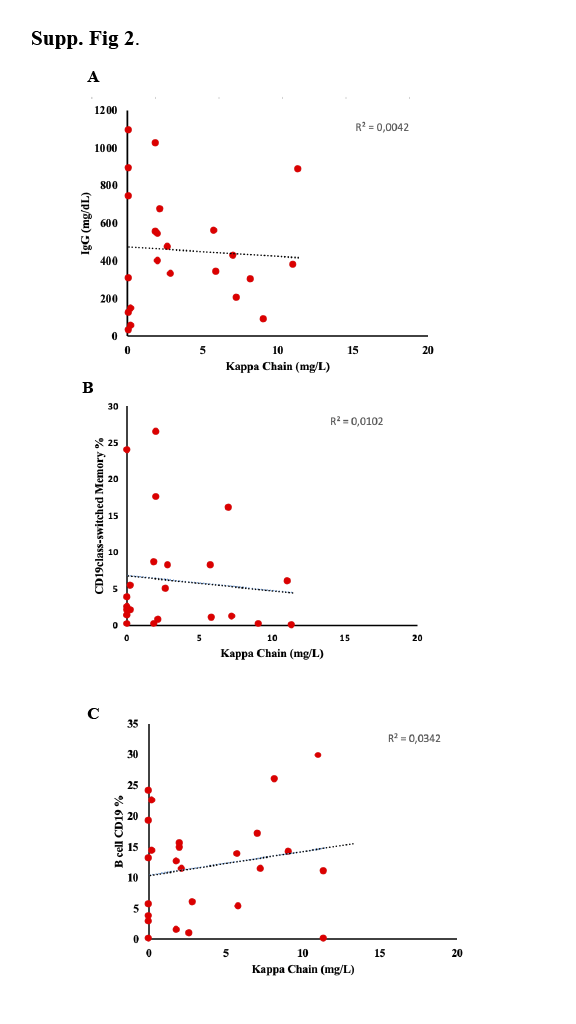

Supplement: FIGURE S2 — Scatter plots comparing Kappa light chain concentration against: (A) Serum IgG at diagnosis; (B) Class-switched Memory B cells (CD19 + IgD-IgM-CD27 +); (C) B cells CD19%. [file Image_2.TIF]

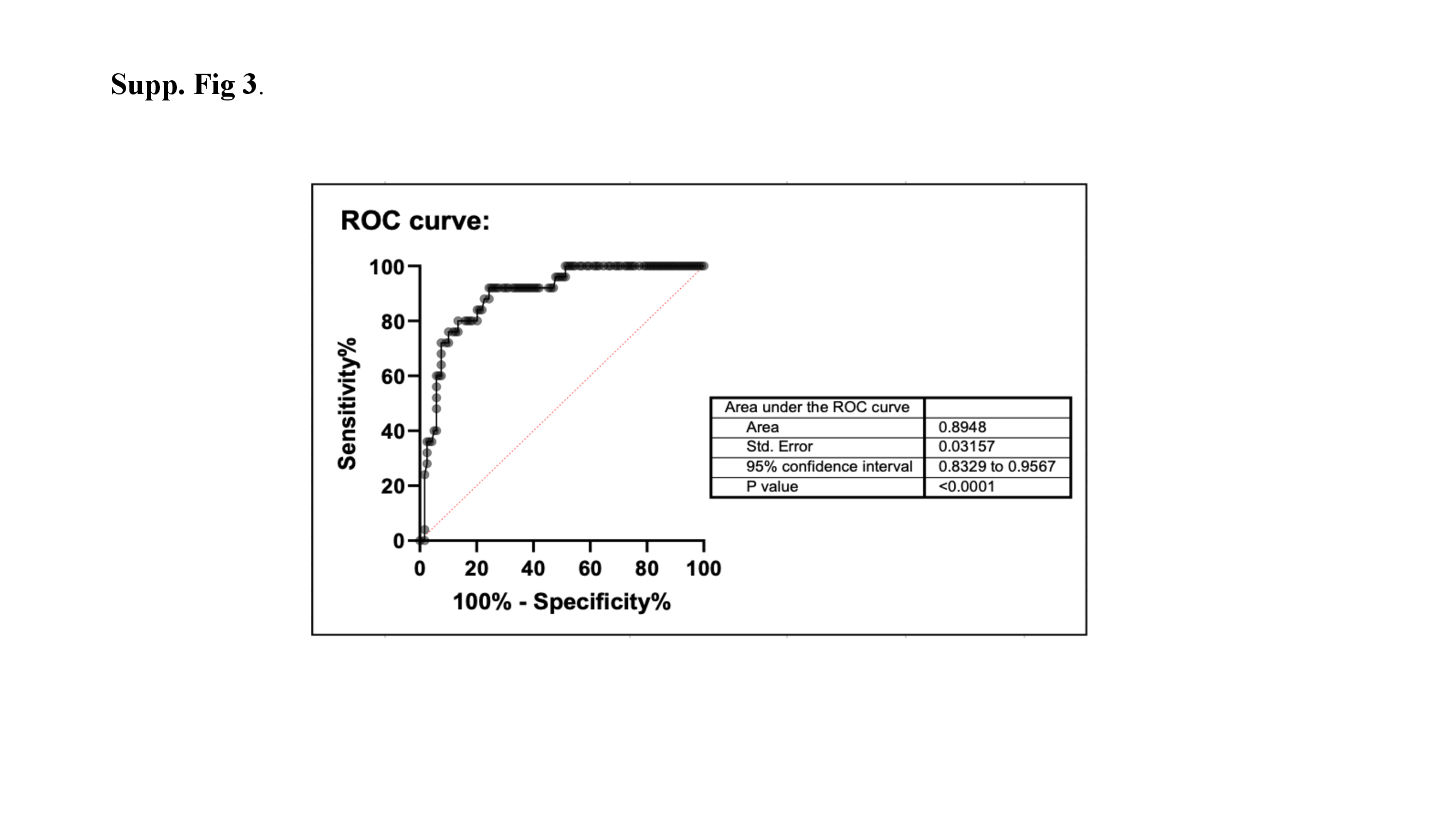

Supplement: FIGURE S3 — Receiver operating characteristic (ROC) curves for CVID diagnosis with the sum κ + λ testing. The area under the curve (AUC) for CVID was 0.894 (P = 0.001). The use of sum κ + λ testing identified 24 of 26 cases of CVID, for a sensitivity of 92% [95% confidence interval (CI), 0.83–0.95 (p < 0.0001)]. [file Image_3.TIFF]
